# Research status and trends of drug-coated balloons in coronary artery disease: a bibliometric analysis

**DOI:** 10.3389/fmed.2025.1591906

**Published:** 2025-08-18

**Authors:** Yue Yu, Yuemiao Jiao, Lüya Wang, Ming Zhang, Chengqian Yin

**Affiliations:** Department of Cardiology, Beijing Anzhen Hospital, Capital Medical University, Beijing, China

**Keywords:** drug-coated balloon, coronary artery disease, percutaneous coronary intervention, bibliometric analysis, research trends

## Abstract

**Objective:**

Drug-coated balloons have emerged as a pivotal alternative to drug-eluting stents in the interventional management of coronary artery disease, particularly showing clinical advantages in the treatment of in-stent restenosis and small-vessel disease. This study provides a systematic bibliometric analysis of publication trends, research hotspots, and future directions in DCB-related CAD research from 2004 to June 2025.

**Methods:**

A total of 1,092 publications indexed in the Web of Science, Scopus, and PubMed databases were analyzed using CiteSpace, VOSviewer, and bibliometrix. Inclusion criteria were English-language papers, while case reports, conference proceedings, news articles, and duplicate publications were excluded. The analysis focused on publication trends, country/institutional contributions, author collaboration networks, journal analysis, co-citation literature, and keyword evolution.

**Results:**

Three distinct developmental phases of DCB research were identified: (1) device optimization (2004–2010), (2) clinical validation (2010–2017), and (3) application to complex lesions (2018–present). China led in publication volume (*n* = 180), while Germany and Italy demonstrated the highest research impact. Leading research institutions included Capital Medical University and Friedrich Schiller University of Jena. High-impact journals such as *JACC: Cardiovascular Interventions* and *EuroIntervention* served as key publication venues, with a focus on clinical outcomes and intravascular imaging. Keyword analysis revealed a growing emphasis on intravascular imaging modalities and emerging drug-coating technologies in recent research.

**Conclusion:**

DCB are now established as a standard of care for ISR (Class IA recommendation), with accumulating evidence supporting their efficacy and safety in small-vessel coronary disease. However, their application in complex lesions requires further validation through multicenter randomized controlled trials. Future research should focus on optimizing drug coating technologies, refining imaging-guided strategies, exploring new anti-proliferative drugs, and establishing more precise eligibility criteria for treatment.

## 1 Introduction

The global rise in cardiovascular disease incidence has rendered it a leading cause of mortality worldwide. Nearly half of adults aged over 20 are affected by cardiovascular diseases, which remain the foremost cause of death globally (1). Coronary artery disease (CAD) constitutes a major subset, posing significant health and economic burdens (2).

Percutaneous coronary intervention (PCI) has evolved through several key stages, including PTCA (percutaneous transluminal coronary angioplasty), BMS (bare-metal stents), DES (drug-eluting stents), and most recently, DCB (drug-coated balloons). DCB integrates the benefits of PTCA (absence of permanent implantation) and DES (anti-proliferative properties), thereby minimizing vascular interference from metallic stents and lowering the risk of late thrombosis. DCB has demonstrated efficacy and safety in treating in-stent restenosis (ISR), and is currently recommended with a Class IA indication (3, 4). Both short- and long-term efficacies of DCB in small-vessel disease have been validated (5); however, robust evidence supporting its application in large vessels and bifurcation lesions remains limited. DCB may reduce bleeding complications in patients with diabetes or high bleeding risk, primarily by limiting the duration of antithrombotic therapy (6). While the study by Tao Ling et al. indicated that DCB combined with bail-out stenting failed to meet the non-inferiority endpoint in newly diagnosed non-complex CAD, prior meta-analyses have yielded inconsistent findings, and DES continues to be the standard of care (7, 8). Additional large-scale randomized controlled trials (RCTs) are warranted to delineate the optimal clinical indications for DCB use.

Bibliometric analysis has emerged as a powerful tool for systematically identifying disciplinary trends and mapping emerging research frontiers. Prior reviews have thoroughly outlined the mechanisms of action, historical evolution, lesion-specific efficacy, and perioperative management strategies of DCB in the treatment of CAD (6, 9). In this study, an extensive dataset integrating the Web of Science, PubMed, and Scopus databases was employed, encompassing a broader and more current timeframe (2004 to June 2025). Advanced clustering techniques were applied using CiteSpace, VOSviewer, and bibliometrix, with results subsequently visualized via Scimago Graphica. Bibliometric approaches enabled quantitative delineation of the evolutionary trajectory, global collaboration networks, and thematic hotspots within the field of DCB research. This analysis provides an objective overview of DCB’s technological evolution and delivers data-driven insights to guide future research in interventional cardiology.

## 2 Materials and methods

### 2.1 Data sources

**Figure 1:**
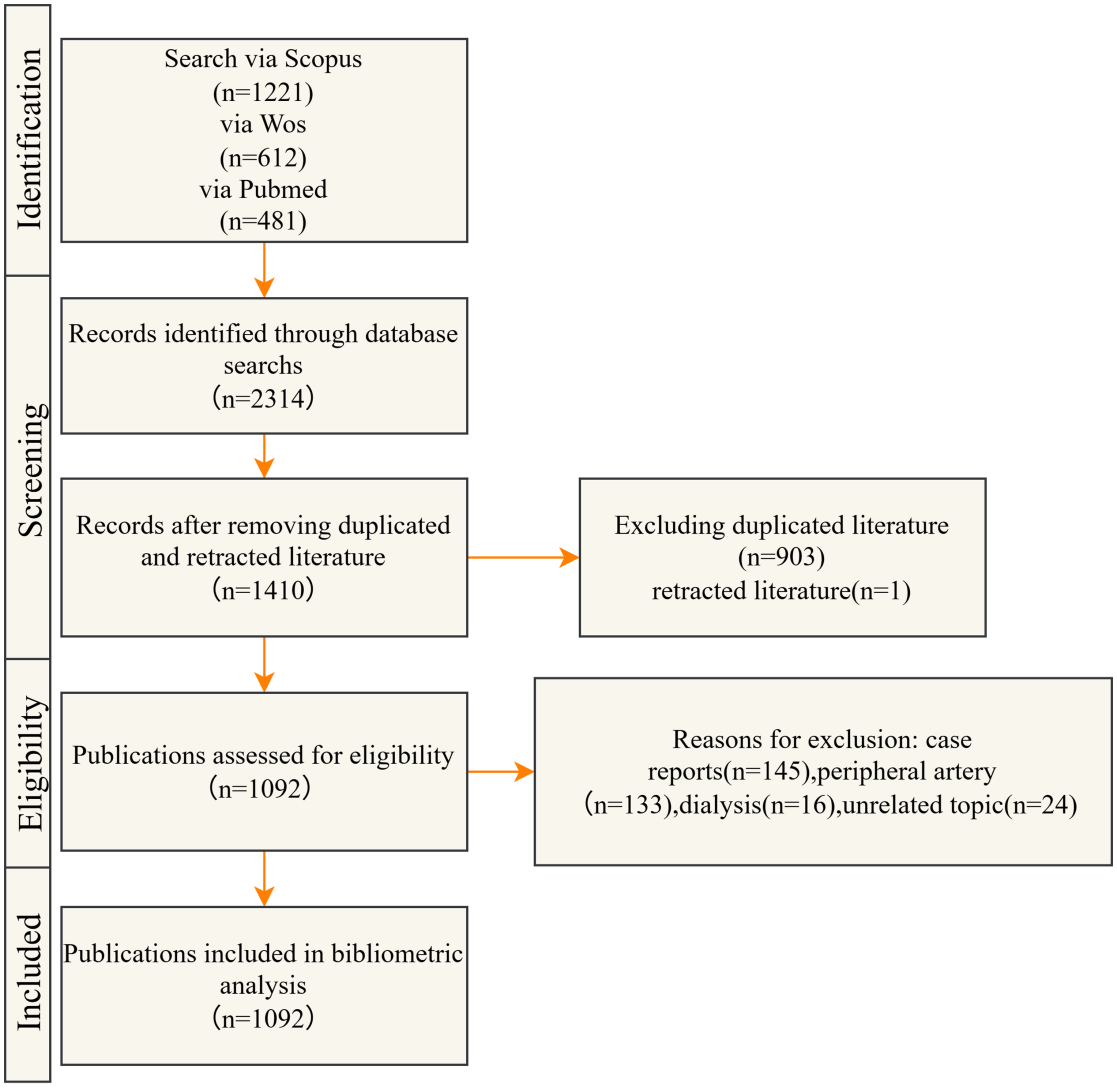
Flowchart of literature search and screening process.

A comprehensive literature search was performed across the Web of Science Core Collection, Scopus, and PubMed databases, encompassing publications from 1 January 2004 to 30 June 2025. Studies investigating the application of DCB in the treatment of CAD were included. Case reports, conference abstracts, news articles, and duplicate records were excluded from the analysis. The detailed screening and selection process is illustrated in Figure 1, and the complete search strategy is outlined in Table 1.

**Table 1:** Keyword search conducted in PubMed, Scopus, and the Web of Science Core Collection database.

Database	Query
WoS	TS = ((“coronary artery disease” OR “coronary heart disease” OR “ischemic heart disease” OR “myocardial ischemia” OR “acute coronary syndrome” OR “angina pectoris” OR “myocardial infarction” OR “CAD” OR “CHD” OR “ACS”) AND (“drug-eluting balloon” OR “drug coated balloon” OR “DEB” OR “DCB” OR “paclitaxel-eluting balloon” OR “sirolimus-eluting balloon” OR “limus-eluting balloon”))
Pubmed	((“coronary artery disease”[Title/Abstract] OR “coronary heart disease”[Title/Abstract] OR “ischemic heart disease”[Title/Abstract] OR “myocardial ischemia”[Title/Abstract] OR “acute coronary syndrome”[Title/Abstract] OR “angina pectoris”[Title/Abstract] OR “myocardial infarction”[Title/Abstract] OR “CAD”[Title/Abstract] OR “CHD”[Title/Abstract] OR “ACS”[Title/Abstract]) AND (“drug-eluting balloon”[Title/Abstract] OR “drug coated balloon”[Title/Abstract] OR “DEB”[Title/Abstract] OR “DCB”[Title/Abstract] OR “paclitaxel-eluting balloon”[Title/Abstract] OR “sirolimus-eluting balloon”[Title/Abstract] OR “limus-eluting balloon”[Title/Abstract]))
Scopus	TITLE-ABS-KEY ((“coronary artery disease” OR “coronary heart disease” OR “ischemic heart disease” OR “myocardial ischemia” OR “acute coronary syndrome” OR “angina pectoris” OR “myocardial infarction” OR “CAD” OR “CHD” OR “ACS”) AND (“drug-eluting balloon” OR “drug coated balloon” OR “DEB” OR “DCB” OR “paclitaxel-eluting balloon” OR “sirolimus-eluting balloon” OR “limus-eluting balloon”))

### 2.2 Analysis methods

Duplicate records retrieved from Scopus, Web of Science, and PubMed were identified using EndNote’s built-in de-duplication function, supplemented with manual verification. Two reviewers (Yu and Jiao) independently screened the retrieved articles, and any discrepancies were resolved by a third reviewer (Yin), who made the final determination. The final dataset was exported in RIS format and subsequently converted to plain text using the built-in converter in CiteSpace (v6.2. R3) (10). The resulting files were named using the format “download_XXX.”

Time slicing was set to one-year intervals, with node selection thresholds adjusted to 25 or 5 depending on specific analytical requirements, as detailed in the main text. Additional parameter settings are described in the main text. Journal and co-citation analyses were conducted using VOSviewer (v1.6.20) (11), and the results were visualized on a global map with Scimago Graphica (v1.0.48) (12). Additionally, a three-field plot linking countries, institutions, and journals was generated using the R package bibliometrix (v4.3.2) (13).

## 3 Results

### 3.1 General information

Based on the predefined inclusion criteria, 2,314 publications were identified, comprising 612 from Web of Science, 1,221 from Scopus, and 481 from PubMed. After applying the exclusion criteria, 1,092 articles were retained for final analysis. The curated dataset was subsequently imported into CiteSpace for further analysis.

A notable increase in publication output was observed beginning in 2011. Although brief declines occurred in 2017 and 2019, publication output rebounded thereafter and has continued to rise annually. As of June 2025, 99 articles had already been retrieved, suggesting that the total for the year may exceed the 129 publications recorded in 2024 (Supplementary Figure 1).

### 3.2 Country/region analysis

A total of 55 countries and 871 institutions contributed publications related to DCB in the context of coronary artery disease. Among these contributors, China produced the highest number of publications (*n* = 180), followed by Germany (*n* = 107), Italy (*n* = 100), and the United States (*n* = 81) (Table 2). In terms of citation impact, Germany and Italy exhibited a clear advantage.

**Table 2:** List of the top 15 countries and regions with the highest research productivity.

Rank	Country	Documents	Citations	Total link strength
1	China	180	1,619	76
2	Germany	107	3,705	160
3	Italy	100	2,041	156
4	USA	81	1,846	137
5	England	54	982	99
6	Japan	50	512	17
7	South Korea	49	871	66
8	Spain	49	1,814	84
9	Netherlands	33	1,104	85
10	Switzerland	29	1,222	72
11	Malaysia	21	751	55
12	Poland	17	95	40
13	Finland	14	688	35
14	France	14	327	46
15	Belgium	13	590	53

**Figure 2:**
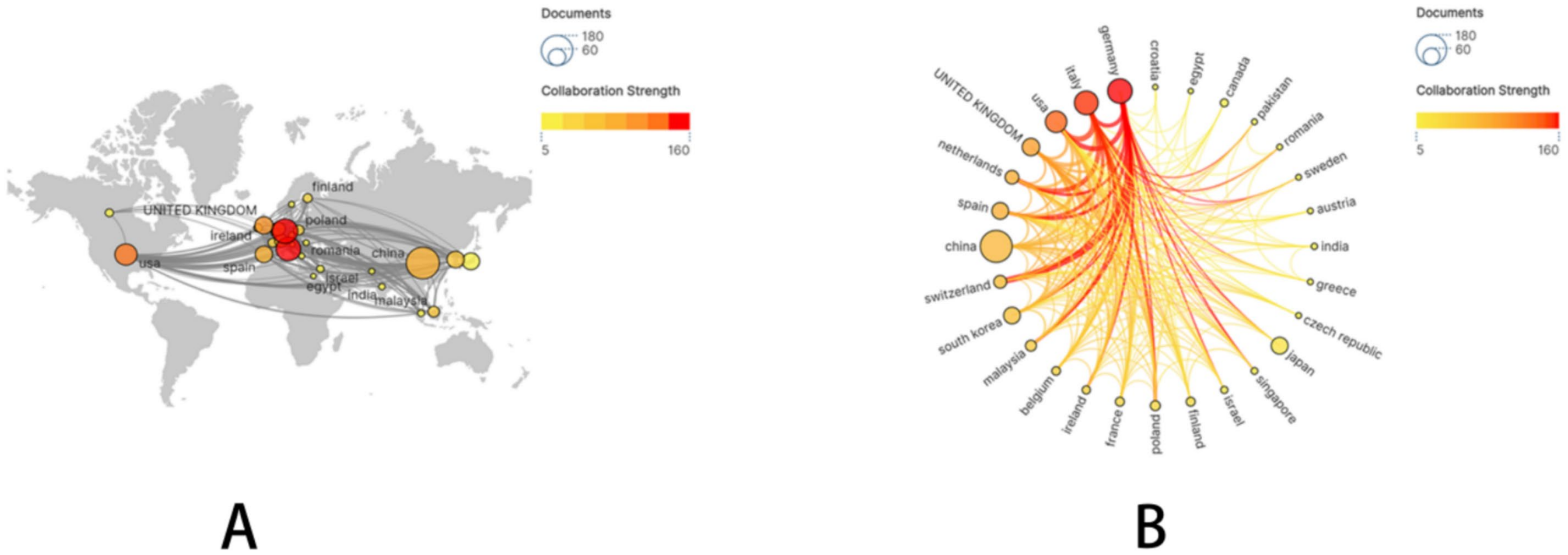
The geographical distribution and visualization of countries on the research of drug-coated balloons in coronary artery disease.

A subset of 28 countries, each with at least five publications, was subsequently selected and visualized. A collaboration network was constructed based on publication volume and inter-country connections (Figure 2). Notably, extensive and active international collaborations were evident. China demonstrated particularly close research collaborations with Germany, the United States, and Italy.

### 3.3 Institutional analysis

An institutional-level analysis of the included publications was conducted using CiteSpace. The K-value was set at 25, and no pruning algorithm was applied. A collaboration network was constructed to visualize inter-institutional relationships (Supplementary Figure 2).

The five most prolific institutions were Capital Medical University (*n* = 28), Friedrich Schiller University of Jena (*n* = 22), Hospital de La Princesa (*n* = 20), University of Ulsan (*n* = 20), and University of Basel (*n* = 20). In terms of betweenness centrality—a key indicator of network influence—the top five institutions were Hospital de La Princesa (0.27), Chinese Academy of Medical Sciences–Peking Union Medical College (0.19), German Heart Centre Munich (0.14), Fu Wai Hospital–CAMS (0.12), and University of Ulsan (0.11).

Collectively, both publication volume and centrality scores underscore the leading role of institutions from China, Germany, South Korea, and Spain, markedly surpassing contributions from other countries (Supplementary Table 1).

### 3.4 Author and co-cited author analysis

**Figure 3:**
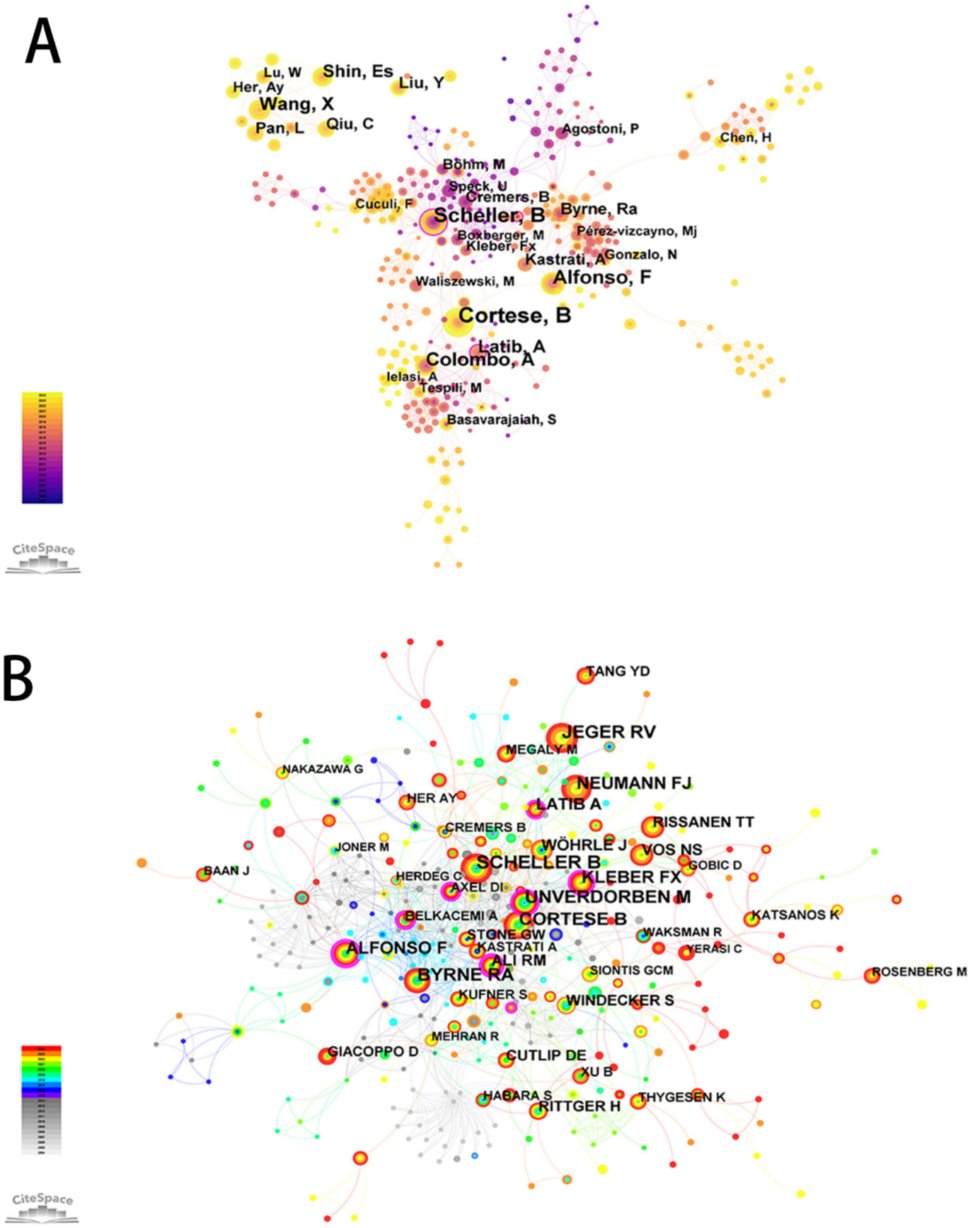
The network map of authors engaged in drug-coated balloons in the field of coronary artery disease. **(A)** Co-authorship Map. **(B)** Co-citation Map.

A co-authorship and co-citation network was constructed using CiteSpace, based on author productivity and citation linkages (Figure 3). The five most prolific authors were Cortese, B (*n* = 74), Scheller, B (*n* = 52), Alfonso, F (*n* = 42), Colombo, A (*n* = 41), and Shin, E.S. (*n* = 30). Authors with the highest betweenness centrality values were Scheller, B (0.31) and Garg, S (0.26) (Supplementary Table 2).

Co-citation analysis revealed the five most frequently co-cited authors as Scheller, B; Cortese, B; Jeger, R.V.; Unverdorben, M; and Byrne, R.A. Additionally, a ranking of authors based on co-citation centrality was presented (Supplementary Table 3).

### 3.5 Journal and co-cited journal analysis

A journal collaboration network was constructed using VOSviewer, based on publication volume and co-citation relationships (Supplementary Figure 3). In the context of coronary artery disease and drug-coated balloon (DCB) research, *Catheterization and Cardiovascular Interventions* (66 publications, Q3) and *EuroIntervention* (31 publications, Q1) emerged as the leading journals by publication output. Notably, *EuroIntervention* exhibited substantial influence, with 1,040 total citations and a co-citation link strength of 53,095, underscoring its prominence in both publication output and scholarly connectivity. *JACC: Cardiovascular Interventions* (Q1) ranked third, with 25 publications, 1,619 total citations (an average of 65 per article), and the highest overall link strength (661), highlighting its academic authority in the field.

In the co-citation rankings, the *Journal of the American College of Cardiology* (Q1) led with 2,178 co-citations and a link strength of 79,118, followed by *Circulation*, *European Heart Journal*, and *The Lancet* (all Q1), Remarkably, Q1 journals constituted 90% of the top 10 most frequently co-cited sources. Notably, *Clinical Research in Cardiology* (Q1) appeared among both the top 10 publishing and co-cited journals, indicating its sustained scholarly relevance in this domain (Supplementary Tables 4, 5).

A journal overlay analysis was performed using CiteSpace, as shown in Supplementary Figure 4. The analysis revealed a “green channel” transition from health/nursing/medicine to medicine/medical/clinical domains, suggesting that DCB research predominantly circulates within clinical medicine and pharmacology under an independent disciplinary model.

In the three-field plot linking countries, institutions, and journals in DCB-related coronary artery disease research (Supplementary Figure 5), Germany was found to have affiliations with 16 institutions, including Friedrich Schiller University of Jena, Humboldt University of Berlin, University of Basel, Charité—Universitätsmedizin Berlin, Technical University of Munich, Medical University of Silesia, German Center for Cardiovascular Research, Berlin Institute of Health, Hospital de La Princesa, German Heart Center Munich, Free University of Berlin, Universitätsklinikum des Saarlandes, University of Ulsan, Università Vita-Salute San Raffaele, Peking Union Medical College, and Fuwai Hospital.

China was associated with 12 institutions, including Capital Medical University, Peking Union Medical College, Fuwai Hospital, Zhengzhou University, Shanghai Jiao Tong University, University of Ulsan, Hospital de La Princesa, German Heart Center Munich, Universitätsklinikum des Saarlandes, German Center for Cardiovascular Research, Technical University of Munich, and University of Basel.

Close institutional collaboration between China and Germany was particularly noteworthy. Notably, Capital Medical University has established links with 10 major journals, including *Catheterization and Cardiovascular Interventions, JACC: Cardiovascular Interventions, Frontiers in Cardiovascular Medicine, Journal of the American College of Cardiology, BMC Cardiovascular Disorders, Journal of Geriatric Cardiology, American Journal of Cardiology, Reviews in Cardiovascular Medicine, Angiology, and Coronary Artery Disease.*

### 3.6 Co-cited references

Co-citation analysis was conducted using CiteSpace, with the K-value set to 25 and no pruning algorithm applied, to visualize co-cited references. Co-cited references in DCB research coronary artery disease research were predominantly published between 2018 and 2020, reflecting growing interest and evidence accumulation in this domain. The most frequently co-cited paper was the study by Jeger et al. (3), published in *JACC: Cardiovascular Interventions* in 2020, with 35 co-citations. Notably, the study by Vos et al. (14), with 27 co-citations, had a high centrality score of 0.1, exhibited a high centrality score of 0.1, indicating its pivotal role within the co-citation network. Additionally, *The Lancet* and *JACC: Cardiovascular Interventions* contributed four and five highly co-cited papers, respectively. Early publications, such as the 2013 study by Byrne et al. (15) (14 co-citations), continue to exert significant influence, highlighting the sustained knowledge continuity in DCB research and positioning this article as a potential seminal work in the field.

The top 10 co-cited references were clustered and visualized along a citation timeline (Supplementary Figure 6). Colored segments represent the periods when initial citation links emerged, with purple denoting earlier citations and yellow representing more recent ones. References positioned on the left of the co-citation network generally correspond to earlier literature. For instance, clusters such as #8 (Updating, 2007), #4 (Pharmacological Prevention, 2007), and #5 (Emerging Applications, 2010) represent early-stage research in the field.

Smaller cluster numbers correspond to larger document counts, suggesting greater thematic importance in DCB research. Citation bursts indicate a rapid increase in citation frequency of specific references over a defined time window. These bursts are highlighted with red circles (Supplementary Figure 6).

Clusters 0, 1, 3, and 4 demonstrated the longest durations, persisting for up to 10 years. Among these, Cluster 0—centered on the drug-coated balloon-only strategy—had the most recent citation activity and included the largest number of documents (*n* = 80). The average citation year was 2019, with a marked increase in co-citation activity observed in recent years. This cluster encompasses several pivotal studies—including the REVELATION trial (14), BASKET-SMALL 2 trial (16), DEBUT trial (17), and PICCOLETO II trial (18), as well as expert consensus and guidelines, such as the *2018 ESC/EACTS Guidelines on Myocardial Revascularization* (19) *and Drug-Coated Balloons for Coronary Artery Disease: Third Report of the International DCB Consensus Group* (3).

Within Cluster 0, the *Third Report of the International DCB Consensus Group* (35 co-citations) was the most frequently co-cited reference. The updated consensus outlined in this document suggested that, in addition to its established use in treating in-stent restenosis in the 2018 ESC guidelines, DCB may also be applicable for primary coronary artery lesions. For example, DCB have shown efficacy and safety in treating *de novo* small vessel disease and may be considered in patients with diabetes or those at high bleeding risk. Furthermore, DCB may also have potential applications in other clinical scenarios, including bifurcation lesions, large vessel disease, and complex coronary interventions. The guideline synthesized existing evidence and highlighted directions for future research.

CiteSpace was also used to identify 10 references exhibiting significant citation bursts (Supplementary Table 7). Citation bursts were visualized using annual bar charts. The strongest citation burst was observed for the article “*Paclitaxel-eluting balloons, paclitaxel-eluting stents, and balloon angioplasty in patients with restenosis after implantation of a drug-eluting stent (ISAR-DESIRE 3): A randomized, open-label trial*” by Robert A. Byrne (15), with a burst strength of 6.35, peaking between 2013 and 2018. The second most intense burst (burst strength = 5) was associated with the paper “*Paclitaxel-coated balloon catheter versus paclitaxel-coated stent for the treatment of coronary in-stent restenosis*” by Martin Unverdorben et al. (20), published in *Circulation*, with a burst period from 2010 to 2014. The citation burst strengths of these 10 references ranged from 3.55 to 6.35, with durations spanning 2 to 6 years.

### 3.7 Keyword analysis

**Figure 4:**
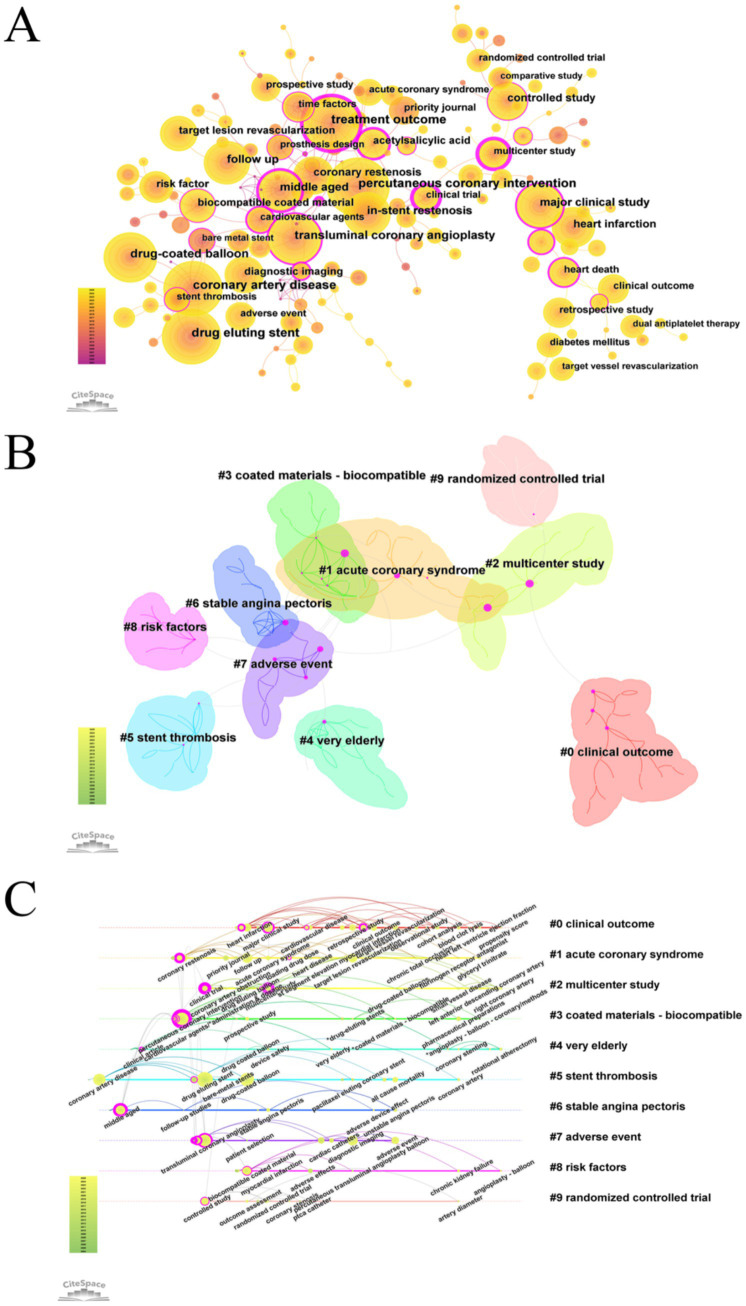
Visualization map of keyword in DCB research for coronary artery disease. **(A)** Keyword co-occurrence map. **(B)** Keyword clustering map. **(C)** Keyword timeline map.

A keyword analysis of studies on drug-coated balloons (DCB) in coronary artery disease over the past two decades was conducted using CiteSpace. A pruning threshold of K = 5 was applied, and semantically similar keywords were consolidated. The top 20 most frequently occurring keywords were identified (Figure 4; Supplementary Figure 7; Supplementary Tables 8, 9).

The most frequent keywords included percutaneous coronary intervention, coronary artery disease, transluminal coronary angioplasty, and drug-coated balloon, ranked first, third, fifth, and sixth, respectively. However, because this study specifically focused on DCB in the context of CAD—and since CAD and DCB were used as search terms, and PCI and PTCA represent standard interventional techniques—these terms were excluded from further keyword analysis. Among the remaining keywords, drug-eluting stent (*n* = 548), treatment outcome (*n* = 519), and in-stent restenosis (*n* = 380) were the most frequently occurring.

However, keyword frequency alone may not adequately reflect research influence. High-frequency keywords with centrality scores ≥ 0.1 often serve as critical nodes within the knowledge network and may serve as partial indicators of research hotspots. Notably, the keywords “treatment outcome” (centrality = 0.69), “major clinical study” (centrality = 0.33), and “middle-aged” (centrality = 0.46) exhibited high centrality, underscoring their pivotal role in shaping the field’s research focus.

The keyword clustering analysis identified 194 nodes and 240 links. The largest connected component consisted of 161 nodes, accounting for 82% of the network. The network modularity was 0.8237, and the average silhouette score was 0.9677, indicating strong internal consistency and well-defined clusters, thereby validating the robustness of the clustering results. Cluster #0 (“clinical outcome”) was the largest, comprising 23 nodes, followed by Cluster #1 (“acute coronary syndrome”) and Cluster #2 (“multicenter study”). These clusters provide insights into distinct research directions and conceptual linkages within the field of drug-coated balloon research in coronary artery disease. The keyword “treatment outcome” exhibited high centrality within both Cluster #1 and Cluster #3 (“coated materials – biocompatible”), highlighting its pivotal role in bridging these thematic areas.

Clusters #9 (“randomized controlled trial”) and #2 (“multicenter study”) represent methodological approaches in clinical study design. The emphasis on multicenter randomized controlled trials underscores the field’s commitment to methodological rigor. Cluster #7 (“adverse event”) underscores the increasing emphasis on the safety profile of DCB. Meanwhile, Cluster #1 (“acute coronary syndromes”) and Cluster #6 (“stable angina pectoris”) reflect distinct clinical subtypes of coronary artery disease, illustrating the field’s increasing stratification and specialization.

Based on the log-likelihood ratio (LLR) analysis, Cluster #0 (“clinical outcome”) included key terms such as “clinical outcome” (LLR = 41.95, *p* < 1.0E–4), “target vessel revascularization” (LLR = 41.52, *p* < 1.0E–4), “bare metal stent” (LLR = 40.04, *p* < 1.0E–4), “retrospective study” (LLR = 38.17, *p* < 1.0E–4), and “adverse effects” (LLR = 37.32, *p* < 1.0E–4). In a study by Mauro Gitto (16), the term target vessel revascularization appeared with high frequency. This study evaluated the use of DCB angioplasty for *de novo* lesions in the left anterior descending (LAD) artery. The findings demonstrated that DCB angioplasty exhibited comparable safety and efficacy to new-generation DES, with a lower two-year target lesion failure rate—potentially attributable to a reduced stent burden. Building upon existing guideline recommendations that support the use of DCB for in-stent restenosis (ISR) and small-vessel disease (3, 19), the study provided new evidence for its application in large-vessel lesions, thereby offering data to inform potential guideline expansion.

A keyword timezone visualization was generated, grouping nodes according to the time periods in which they emerged, thereby facilitating the identification of emerging research trends (Figure 4). This analysis revealed that 2016 marked the emergence of terms such as “OCT” and “intravascular ultrasound,” signaling the rise of intravascular imaging–based precision assessments. In the same year, Fadi J. Sawaya et al. (21) published a review entitled *Contemporary Approach to Coronary Bifurcation Lesion Treatment*, which comprehensively outlined strategies for managing bifurcation lesions. The article discussed the potential application of DCB in side-branch treatment—while acknowledging the limited supporting evidence and the need for further investigation—and advocated for the use of intravascular imaging to refine Medina classification and guide clinical decision-making.

The evolution of research themes in DCB-related studies from 2004 to June 2025 was mapped, revealing dynamic shifts in keyword prominence and thematic focus. Early investigations (2004–2010) focused primarily on device engineering, encompassing balloon catheter technologies and comparisons with bare-metal stents. Between 2010 and 2017, research emphasis shifted toward drug-eluting balloons and comparative studies with DES, particularly those involving paclitaxel and everolimus. More recent investigations have focused on clinical outcomes and adverse events (2014–2017), as well as procedural optimization (2015–2020). Overall, the research trajectory has progressed from foundational device development toward advanced drug delivery platforms, clinical efficacy evaluation, and safety profiling—reflecting continued technological innovation and increasing clinical relevance within the field.

## 4 Discussion

In recent years, drug-coated balloons (DCB) have emerged as a pivotal innovation in interventional cardiology for the treatment of coronary artery disease (CAD), gradually evolving into a major research focus. This study systematically investigated the research landscape and temporal trends of DCB use in CAD from 2004 to June 2025 using bibliometric approaches. The findings elucidate the developmental trajectory, patterns of international collaboration, key research contributors, and prospective research directions, offering valuable insights for both academic research and clinical application.

The present work complements and resonates with recent comprehensive reviews by Shahrori et al. and Bhogal et al. (22, 23) on the application of DCB in the management of coronary artery disease. From the perspectives of scope and methodology, this study employed bibliometric techniques to elucidate the developmental trajectory and evolving research trends of DCB from a macro-level standpoint. In contrast, the aforementioned reviews systematically examined DCB classification, underlying mechanisms, and pivotal clinical trials from a micro-level perspective. Collectively, these complementary efforts contribute to a more comprehensive and cohesive body of evidence in the field.

Both reviews consistently identified DCB as an effective therapeutic strategy for ISR, a recommendation endorsed by the 2018 European Society of Cardiology (ESC) guidelines with a Class IA level of evidence (4). Bhogal et al. comprehensively reviewed randomized controlled trials (e.g., PACCOCATH, PEPCAD II, ISAR-DESIRE III), highlighting that DCB are either superior or non-inferior to second-generation DES in reducing target lesion revascularization (TLR) and major adverse cardiovascular events (MACE). Moreover, the advantage of shortened dual antiplatelet therapy (DAPT) duration renders DCB especially beneficial for patients with high bleeding risk. This conclusion was reinforced by Cortese’s group, who further observed regional discrepancies in guideline recommendations: while European guidelines favor DCB, U.S. Guidelines tend to advocate repeat DES implantation (24). These observations are consistent with our bibliometric findings, wherein the keyword “in-stent restenosis” has appeared with high frequency (*n* = 380) since 2010, highlighting ISR as a persistent and central focus in DCB-related research.

The development of DCB has transitioned from engineering design to clinical validation. Early investigations (2004–2010) focused primarily on balloon catheter design, drug-release kinetics, and comparative analyses with BMS. This trend is reflected by the frequent emergence of keywords such as “equipment design” and “balloon catheter.” Following the successful integration of anti-proliferative agents such as paclitaxel, the clinical efficacy of DCB in treating ISR and small-vessel disease has been progressively validated. After 2010, research focus shifted toward evaluating clinical outcomes, as evidenced by the rising centrality of keywords such as “treatment outcome” and “adverse effects.” In recent years, the integration of intravascular imaging modalities—such as optical coherence tomography (OCT) and intravascular ultrasound (IVUS)—has significantly advanced precision-guided therapy. However, current evidence remains limited. Although multiple guidelines support the use of DCB for ISR (Class IA evidence) (3, 4), their indications in large-vessel and bifurcation lesions require further validation through rigorous randomized controlled trials. Notably, recent findings from multicenter RCTs contradict earlier meta-analyses, underscoring heterogeneity in research outcomes and existing gaps in the evidence base. Cortese’s review included an evaluation of the ULTIMATE III trial (25), which demonstrated that IVUS-guided intervention significantly improved late-lumen outcomes and procedural efficacy compared to angiography-based strategies. These findings reinforce the role of IVUS in enhancing the therapeutic efficacy of DCB. Similarly, Bhogal et al. emphasized that recent research has increasingly focused on the impact of intravascular imaging on DCB efficacy, suggesting a promising direction for integrating DCB therapy with advanced intracoronary imaging techniques.

Over the course of DCB development, several landmark clinical trials have shaped the research trajectory and significantly influenced bibliometric patterns in the field. Martin Unverdorben et al. (20) published the PEPCAD II trial in *Circulation*, which compared the efficacy of paclitaxel-coated balloons with paclitaxel-eluting stents for treating ISR. The trial concluded that paclitaxel-coated balloons offered comparable efficacy while demonstrating superiority in key angiographic outcomes. These findings underscored the principle that effective restenosis inhibition does not necessarily require the implantation of an additional stent. Detlef G. Mathey et al. (26) published the PEPCAD V study, providing early evidence for the safety and efficacy of DCB in the treatment of side-branch lesions. Raban V. Jeger et al. (16) conducted the pivotal BASKET-SMALL 2 trial, which focused on therapeutic strategies for small coronary vessels (diameter < 3 mm). Although second-generation DES had become the standard of care for coronary artery disease, their performance in small-vessel lesions remained suboptimal, with relatively high rates of adverse events. Although DCB had shown efficacy in ISR—including both bare-metal stent and DES failures—their role in *de novo* small-vessel disease remained unsupported by robust randomized evidence. This large-scale randomized controlled trial enrolled 758 patients with successfully pre-dilated lesions and, using a rigorous design, demonstrated that DCB were non-inferior to second-generation DES in terms of MACE at 12 months. The trial emphasized that when satisfactory angiographic outcomes are achieved after pre-dilatation, DCB therapy for small-vessel coronary disease is both safe and effective. Notably, subgroup analyses yielded consistent results without evidence of heterogeneity, further reinforcing the robustness of the conclusions. The novelty of this trial lies in its expansion of DCB indications through high-quality evidence, thereby offering a new therapeutic option for small-vessel coronary artery disease. Its findings not only offered valuable clinical guidance but also informed the design and conduct of subsequent investigations. These key trials were all cited in the *Third Report of the International DCB Consensus Group* (2020), which subsequently informed updates to relevant clinical guidelines. Collectively, these trials have established a continuum of evidence supporting the expanded use of DCB—from ISR management to *de novo* coronary lesions—ultimately presenting a novel interventional paradigm for coronary artery disease.

From a national perspective, China leads in absolute publication volume with 180 articles; however, Germany and Italy demonstrate greater academic influence, as indicated by higher total citation counts (3,705 and 2,041, respectively) and centrality values (169 and 156), suggesting superior research quality. The team led by Runlin Gao in China evaluated the efficacy of DCB versus DES for small vessel disease and demonstrated that DCB was non-inferior to DES with respect to both efficacy and safety (27). YaLing Han and colleagues investigated a novel DCB device coated with BA9—a semi-synthetic sirolimus analog characterized by enhanced lipophilicity and optimized balloon-based drug delivery. Their findings demonstrated superior efficacy compared to plain old balloon angioplasty (POBA), and future research may explore head-to-head comparisons with DES or alternative DCB formulations (28). Both studies targeted small vessel disease, a clinical area not yet clearly defined in existing guidelines, thereby contributing valuable evidence to support the potential expansion of DCB indications. Institutional analysis identified Capital Medical University (28 publications), Friedrich Schiller University of Jena (22), and Hospital de La Princesa (20) as key contributors. Notably, Hospital de La Princesa (centrality = 0.27) and Peking Union Medical College (centrality = 0.19) occupied central nodes in the institutional collaboration network. Notably, inter-institutional collaboration at the international level remains robust (16, 29). A study by Daniele Giacoppo et al. comparing the efficacy of DCB versus DES for ISR lesions integrated patient-level data from multiple centers across Germany, the United States, Ireland, Spain, Belgium, South Korea, China, and the Netherlands. The study concluded that DCB was superior to repeat DES implantation in treating BMS-ISR lesions, whereas repeat DES was preferable for DES-ISR lesions. This research contributed to refining ISR lesion classification and offered evidence-based guidance for selecting optimal treatment devices (30). Multicenter studies, by enabling parallel data collection across geographically diverse sites, facilitate rapid patient recruitment, enhance sample representativeness and statistical power, and improve the generalizability and reliability of research findings. Moreover, multicenter designs promote resource sharing and cross-institutional collaboration, thereby accelerating research progress and enhancing the overall quality of clinical investigations.

At the author level, Cortese B (74 publications) and Scheller B (52) emerged as the most prolific contributors, with Scheller also occupying a central position in the co-authorship network (centrality = 0.31). Among highly cited authors, Scheller B (71 citations), Cortese B (60), Jeger R.V. (59), Unverdorben M (53), and Byrne R.A. (50) have collectively established the evidence-based foundation for DCB therapy. Notably, the ISAR-DESIRE 3 trial by Byrne R.A., published in 2013 (15), compared DCB, DES and POBA for the treatment of DES-ISR. The results demonstrated that both DCB and DES were superior to POBA, supporting the clinical utility of DCB for managing DES-ISR. The study exhibited a citation burst strength of 6.35, marking it as a seminal work in the field. Scheller B’s research spans multiple dimensions of DCB therapy, encompassing lesion types such as ISR (31), small vessel disease (32), NSTEMI (33), and *de novo* lesions (34), in addition to comparative studies on various drug coatings (35). These core authors have focused on comparative efficacy, long-term safety, and indication expansion, collectively shaping updates to clinical guidelines.

Journal-level analysis reveals that DCB research is predominantly published in Q1-ranked journals. Among these, *JACC: Cardiovascular Interventions* (IF = 11) has published 25 articles, garnering 1,619 citations—an average of 65 citations per article—underscoring its scientific authority in the field. Its published studies primarily address indication expansion (31, 36–38) and intraoperative imaging guidance (25). Although *EuroIntervention* (IF = 9.5) has a slightly lower publication volume (31 articles), it has accumulated 1,040 citations, reflecting substantial influence on clinical practice. Among co-cited journals, *J Am Coll Cardiol* (2,178 citations) and *Circulation* (2,035 citations) form a high-impact cluster, indicating that DCB research is tightly integrated with leading cardiovascular publications. Furthermore, dual-map overlay analysis reveals that DCB research is concentrated at the interface between clinical medicine and pharmacology. The observed pattern of “internal circulation” in knowledge dissemination underscores the need for greater interdisciplinary integration, particularly with materials science and bioengineering.

The keyword time-zone map reveals that DCB research has evolved through three distinct phases: an early phase (2004–2010) focused on device optimization, a transitional phase (2010–2017) centered on efficacy comparisons with DES, and a recent phase (post-2018) characterized by increasing emphasis on complex lesions (e.g., bifurcation lesions), high-risk populations (e.g., patients with diabetes), and long-term safety evaluation. Cluster analysis further identifies “clinical outcome” and “stent thrombosis” as recent research hotspots, underscoring persistent concerns regarding the long-term safety of DCB. For instance, Simone Fezzi et al. (5) reported that DCB significantly reduce the incidence of MACE compared to DES in the treatment of small vessel disease. Nonetheless, several challenges persist. First, regarding indication expansion, although international consensus statements support the potential use of DCB in *de novo* lesions, robust evidence from large-scale RCTs remains scarce. Second, in terms of technical refinement, drug-coating technologies require further optimization to enhance therapeutic efficacy. Recent meta-analyses have explored the comparative efficacy of various drug formulations in patients with CAD. Ramy Sedhom et al. (39) compared limus-coated and paclitaxel-coated DCBs, reporting no significant clinical differences, although paclitaxel-coated balloons were associated with superior imaging outcomes. In a study specifically targeting ISR, Haiwei Liu et al. (40) compared the efficacy of sirolimus-coated versus paclitaxel-coated balloons. Their findings indicated that sirolimus-coated balloons were non-inferior to paclitaxel-coated counterparts with respect to late lumen loss (LLL) at 9 months. However, head-to-head randomized controlled trials comparing sirolimus- and paclitaxel-coated DCB remain limited by small sample sizes and inadequate statistical power. Further investigations are warranted to assess the safety and efficacy of novel drug coatings and to identify potentially superior therapeutic strategies. Moreover, substantial heterogeneity across existing studies highlights the need for high-quality original research and updated meta-analyses to generate a more unified body of evidence.

It is important to recognize that the clinical application of DCB varies substantially across vascular territories and procedural contexts. Although our analysis focuses on coronary artery disease, DCB have gained broader acceptance in peripheral arterial interventions, particularly for femoropopliteal lesions. These lesions are prone to restenosis following plain balloon angioplasty, while stent implantation in long or tortuous segments is often suboptimal, making DCB the preferred therapeutic alternative (41–43). Emerging evidence also suggests potential advantages of DCB in the treatment of intracranial arterial stenosis (44–47). However, this application remains underexplored due to the small caliber and fragile nature of the target vessels, which present considerable technical challenges.

In contrast, within coronary interventions, DCB are predominantly considered a second-line strategy (3, 6). DES remain the first-line option for most *de novo* lesions, owing to their procedural efficiency and high immediate success rates. This discrepancy between bibliometric trends and real-world clinical practice highlights the need to distinguish research focus from clinical adoption. Future studies should not only assess the efficacy of DCB, but also elucidate the barriers hindering their broader implementation across different vascular domains.

## 5 Limitations

While bibliometric analysis provides valuable insights into research trends, several inherent limitations warrant consideration. First, language bias may have occurred, as this study primarily included English-language publications indexed in major databases (Web of Science, Scopus, and PubMed), potentially excluding relevant studies published in other languages. Second, citation behavior may confound the results: high citation counts can reflect academic influence rather than clinical relevance, while practices such as self-citation or preferential citation within research networks may artificially inflate specific metrics. Third, database coverage bias may affect the comprehensiveness of the analysis, as certain journals and conference proceedings might not be indexed by the selected databases. Additionally, citation time-lag may lead to underrepresentation of recent publications, whose academic impact has not yet fully materialized.

These limitations indicate that, although bibliometric approaches are powerful tools for mapping scientific landscapes, their findings should be interpreted alongside qualitative reviews and clinical evidence. Future investigations may benefit from integrating non-English databases (e.g., CNKI), applying machine learning techniques to refine keyword clustering, and extending the observation window to more comprehensively capture temporal dynamics within the field.

## 6 Conclusion

Drug-coated balloon (DCB) therapy has become an increasingly important modality in the percutaneous treatment of coronary artery disease, offering a non-implant, antiproliferative alternative to stent-based strategies. The evolution of DCB research can be broadly categorized into three phases: an initial phase (2004–2010) focused on device development and pharmacokinetics; an intermediate phase (2010–2017) centered on clinical efficacy and safety validation; and a contemporary phase (2018–present) marked by expanding application to complex lesions and high-risk patient subsets. Current guideline-endorsed evidence (Class IA) supports the use of DCB in treating in-stent restenosis, and accumulating data suggest non-inferiority to drug-eluting stents (DES) in small-vessel disease. However, for more challenging scenarios—such as large-vessel, bifurcation, and calcified lesions—robust evidence from large-scale randomized trials remains limited. Future research should focus on optimizing drug-delivery technologies, integrating advanced imaging guidance, exploring next-generation antiproliferative agents such as sirolimus analogs, and refining patient selection criteria. Large-scale, multicenter randomized studies with extended follow-up will be essential to defining the broader clinical role of DCB.

## Data Availability

The original contributions presented in the study are included in the article/Supplementary material, further inquiries can be directed to the corresponding authors.
