# Supplementary material for: Research status and trends of drug-coated balloons in coronary artery disease: a bibliometric analysis
Source: Front Med (Lausanne). 2025 Aug 18;12:1591906. doi: 10.3389/fmed.2025.1591906 (PMC12399631; doi:10.3389/fmed.2025.1591906)
Supplement: Supplementary file 1 [file Supplementary_file_1.docx]

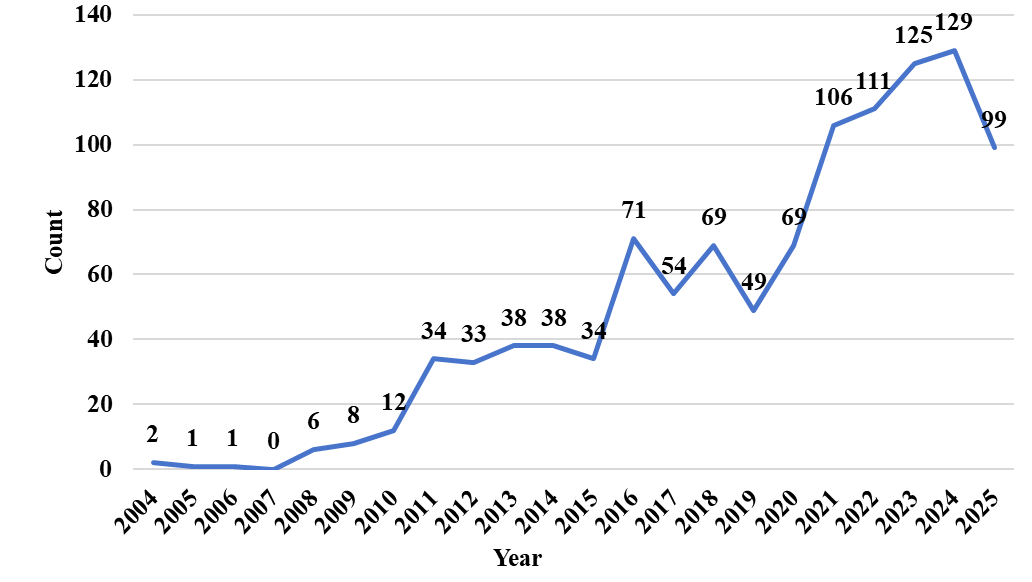


Supplementary Figure 1 Annual Publications of DCB related Studies in the Coronary Heart Disease from 2004 to 2025.


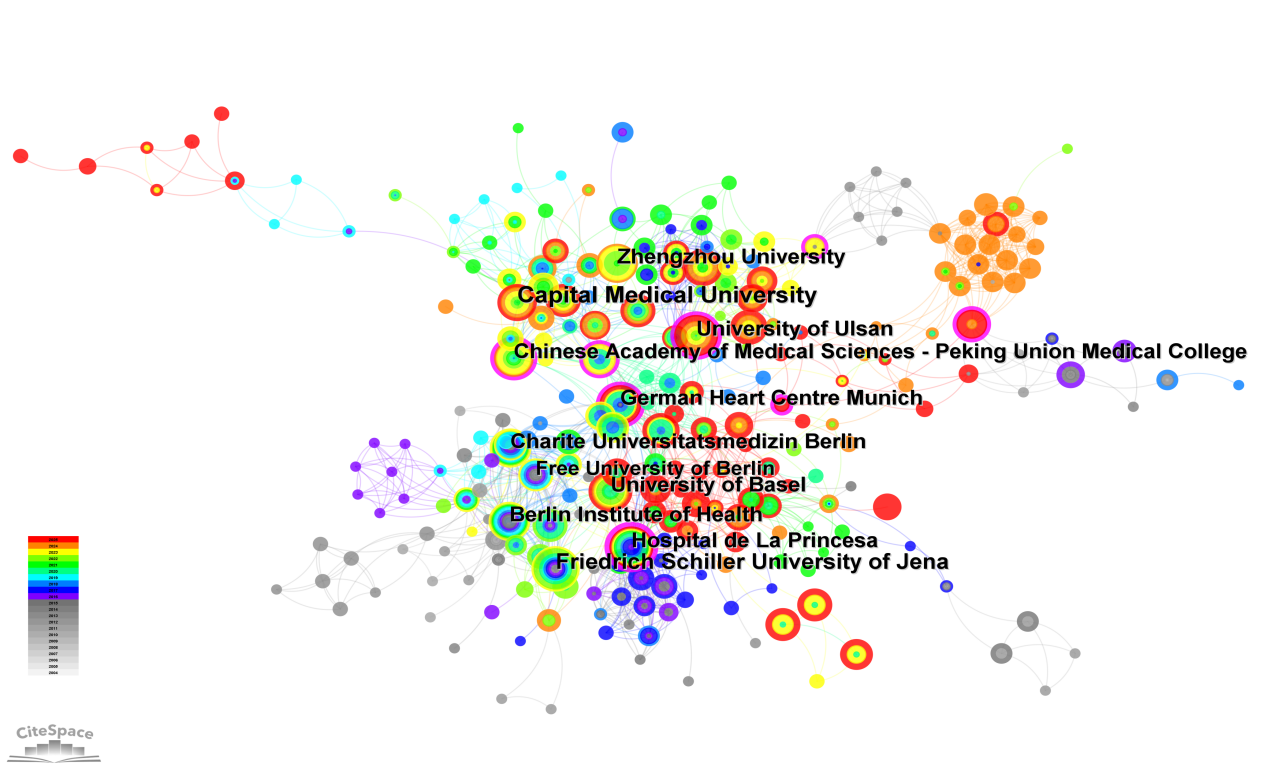


Supplementary Figure 2 The institutional visualization map.

Supplementary Table 1 The top 10 institutions by the number of published articles and centrality ranking.

| Rank | Institution | Counts | Rank | Institution | Centrality |
| --- | --- | --- | --- | --- | --- |
| 1 | Capital Medical University | 28 | 1 | Hospital de La Princesa | 0.27 |
| 2 | Friedrich Schiller University of Jena | 22 | 2 | Chinese Academy of Medical Sciences - Peking Union Medical College | 0.19 |
| 3 | Hospital de La Princesa | 20 | 3 | German Heart Centre Munich | 0.14 |
| 4 | University of Ulsan | 20 | 4 | Fu Wai Hospital - CAMS | 0.12 |
| 5 | University of Basel | 20 | 5 | University of Ulsan | 0.11 |
| 6 | German Heart Centre Munich | 19 | 6 | Gyeongsang National University | 0.11 |
| 7 | Charite Universitatsmedizin Berlin | 19 | 7 | University of Catania | 0.1 |
| 8 | Berlin Institute of Health | 19 | 8 | Autonomous University of Madrid | 0.1 |
| 9 | Chinese Academy of Medical Sciences - Peking Union Medical College | 18 | 9 | Asan Medical Center | 0.1 |
| 10 | Zhengzhou University | 18 | 10 | Royal College of Surgeons - Ireland | 0.1 |

Supplementary Table 2 The top 10 authors by the number of published articles and centrality ranking.

| Rank | Authors | Counts | Country | Rank | Authors | Centrality | Country |
| --- | --- | --- | --- | --- | --- | --- | --- |
| 1 | Cortese, B | 74 | America | 1 | Scheller, B | 0.31 | Germany |
| 2 | Scheller, B | 52 | Germany | 2 | Garg, S | 0.26 | UK |
| 3 | Alfonso, F | 42 | Spain | 3 | Alfonso, F | 0.15 | Spain |
| 4 | Colombo, A | 41 | Italy | 4 | Latib, A | 0.11 | America |
| 5 | Shin, Es | 30 | Korea | 5 | Zhou, Y | 0.1 | China |
| 6 | Latib, A | 29 | America | 6 | Colombo, A | 0.09 | Italy |
| 7 | Wang, X | 29 | China | 7 | Naber, C | 0.08 | Germany |
| 8 | Liu, Y | 21 | China | 8 | Li, X | 0.07 | China |
| 9 | Byrne, Ra | 19 | Ireland | 9 | Zhang, R | 0.07 | China |
| 10 | Her, Ay | 18 | Korea | 10 | Basavarajaiah, S | 0.05 | UK |

Supplementary Table 3 The top 10 Cited authors by the number of articles and centrality ranking.

| Rank | Cited author | Counts | Country | Rank | Cited author | Centrality | Country |
| --- | --- | --- | --- | --- | --- | --- | --- |
| 1 | SCHELLER B | 71 | Germany | 1 | ALFONSO F | 0.25 | Spain |
| 2 | CORTESE B | 60 | America | 2 | ALI RM | 0.23 | Malaysia |
| 3 | JEGER RV | 59 | Switzerland | 3 | UNVERDORBEN M | 0.19 | America |
| 4 | UNVERDORBEN M | 53 | America | 4 | AXEL DI | 0.17 | Germany |
| 5 | BYRNE RA | 50 | Ireland | 5 | KLEBER FX | 0.15 | Germany |
| 6 | NEUMANN FJ | 46 | Germany | 6 | LATIB A | 0.11 | America |
| 7 | KLEBER FX | 45 | Germany | 7 | KASTRATI A | 0.09 | Germany |
| 8 | ALFONSO F | 45 | Spain | 8 | CORTESE B | 0.05 | America |
| 9 | LATIB A | 37 | America | 9 | BYRNE RA | 0.05 | Ireland |
| 10 | ALI RM | 31 | Malaysia | 10 | STONE GW | 0.05 | America |


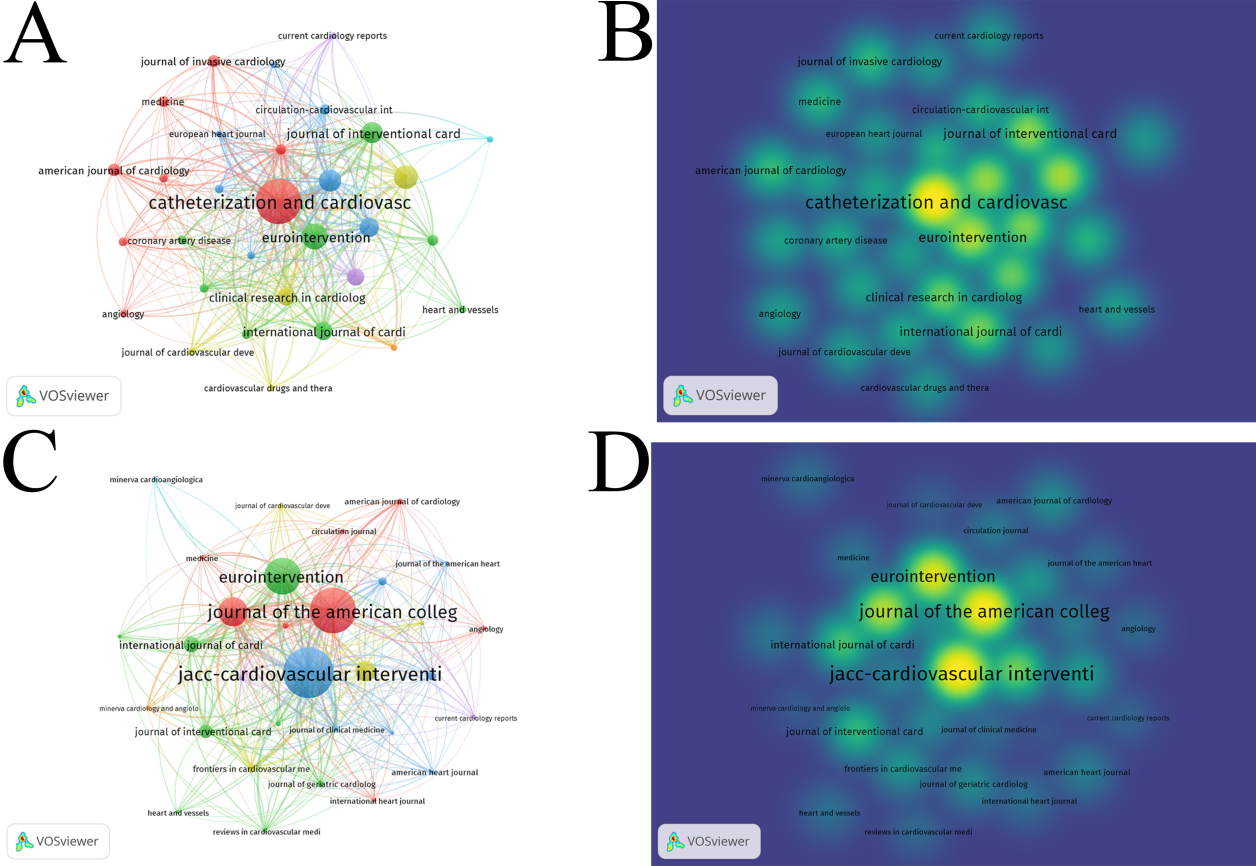


Supplementary Figure 3 Distribution of publications of journals in drug-coated balloon (DCB) research for coronary artery disease.(A) Distribution of journal publications. (B) Heatmap of journal publication volume. (C) Distribution of journal citations. (D) Heatmap of journal citation impact. The size of nodes represents the volume of publications or citations, and brighter colors indicate higher citation counts.

Supplementary Table 4 Top 10 journals by the number of publications

| Rank | source | documents | citations | total link strength | IF(2024) | JCR(2024) |
| --- | --- | --- | --- | --- | --- | --- |
| 1 | catheterization and cardiovascular interventions | 66 | 763 | 528 | 1.9 | Q3 |
| 2 | eurointervention | 31 | 1040 | 417 | 9.5 | Q1 |
| 3 | frontiers in cardiovascular medicine | 28 | 122 | 305 | 2.9 | Q2 |
| 4 | jacc-cardiovascular interventions | 25 | 1619 | 661 | 11.4 | Q1 |
| 5 | journal of interventional cardiology | 24 | 275 | 203 | 1.7 | Q3 |
| 6 | journal of clinical medicine | 21 | 32 | 232 | 2.9 | Q1 |
| 7 | international journal of cardiology | 20 | 335 | 209 | 3.2 | Q2 |
| 8 | clinical research in cardiology | 19 | 476 | 256 | 3.7 | Q1 |
| 9 | bmc cardiovascular disorders | 18 | 67 | 126 | 2.3 | Q2 |
| 10 | american journal of cardiology | 12 | 95 | 93 | 2.1 | Q3 |

Supplementary Table 5 Top 10 journals by co-citation frequency.

| Rank | source | citations | total link strength | IF(2024) | JCR(2024) |
| --- | --- | --- | --- | --- | --- |
| 1 | j am coll cardiol | 2178 | 79118 | 22.3 | Q1 |
| 2 | jacc-cardiovascular interventions | 2035 | 75289 | 11 | Q1 |
| 3 | circulation | 1660 | 64600 | 38.6 | Q1 |
| 4 | eurointervention | 1377 | 53095 | 9.5 | Q1 |
| 5 | catheter cardio inte | 967 | 39946 | 1.9 | Q3 |
| 6 | eur heart j | 967 | 36893 | 35.6 | Q1 |
| 7 | lancet | 881 | 34997 | 88.5 | Q1 |
| 8 | new engl j med | 826 | 33748 | 78.5 | Q1 |
| 9 | clinical research in cardiology | 680 | 25513 | 3.7 | Q1 |
| 10 | circ-cardiovasc inte | 547 | 22061 | 7.4 | Q1 |


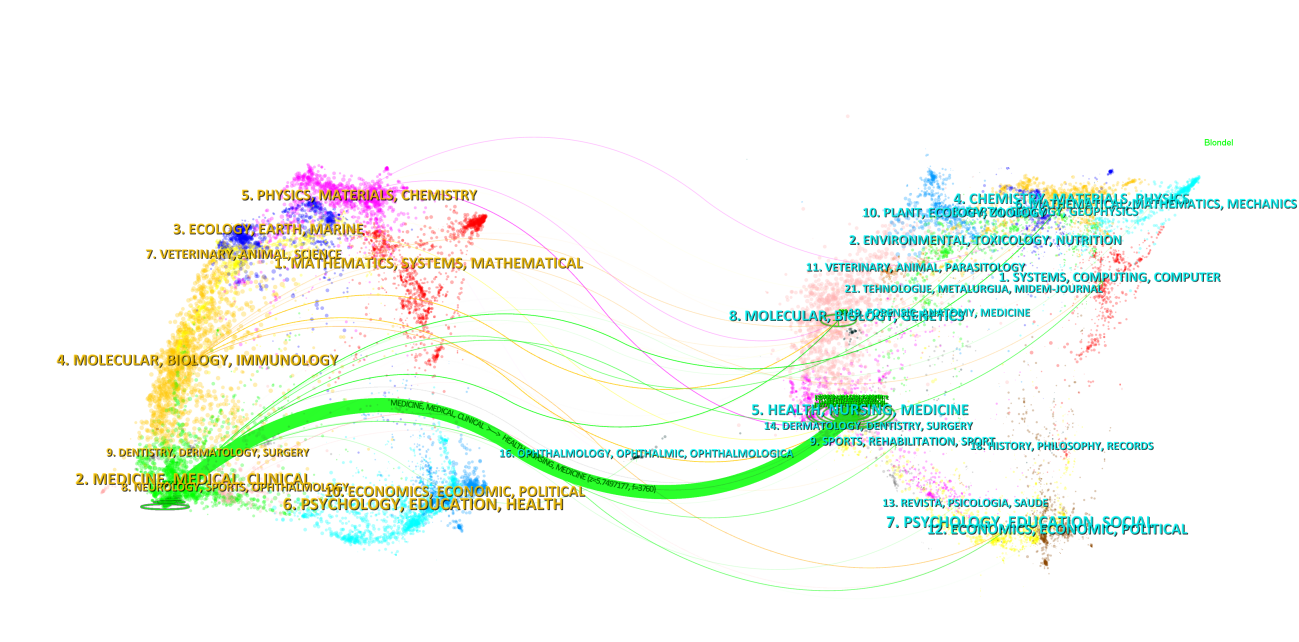


Supplementary Figure 4 Dual-map overlay of journals in DCB research for coronary artery disease.


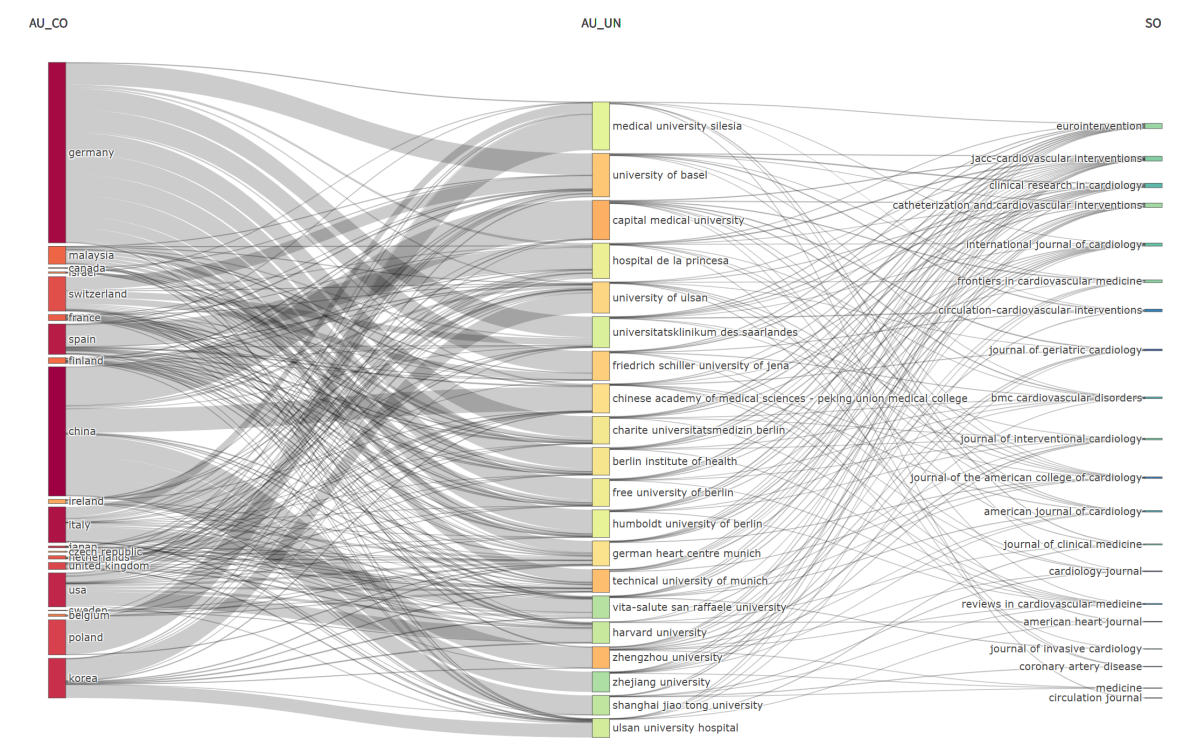


Supplementary Figure 5 Three-field plot of countries, institutions, and journals in DCB research for coronary artery disease.


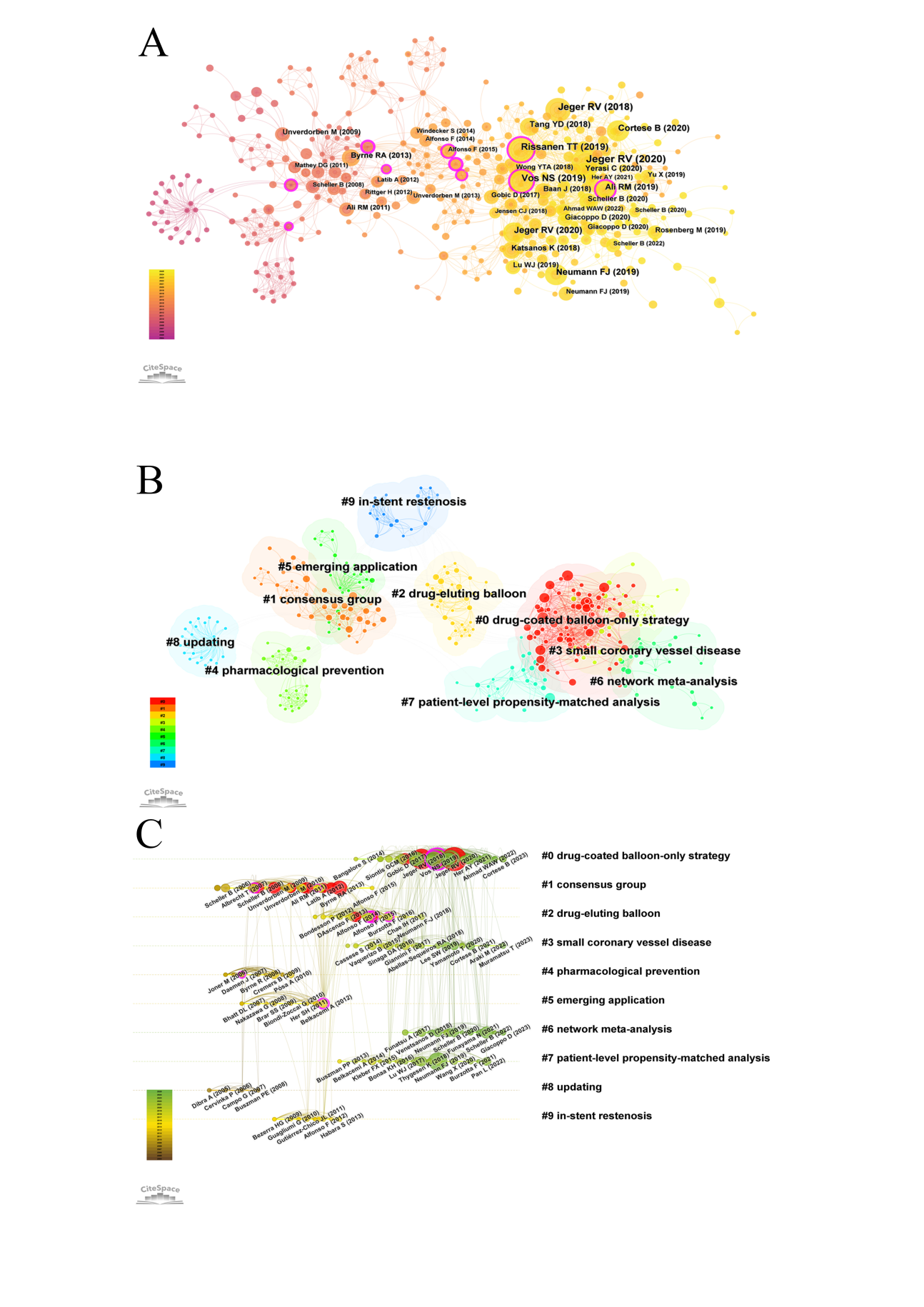


Supplementary Figure 6 Visualization map of reference co-citation network in DCB research for coronary artery disease.

Supplementary Table 6 Top 10 co-cited references in DCB research for coronary artery disease.

| Rank | Cited reference | Year | Counts | Centrality |
| --- | --- | --- | --- | --- |
| 1 | Jeger RV, 2020, JACC-CARDIOVASC INTE, V13, P1391, DOI 10.1016/j.jcin.2020.02.043[1] | 2020 | 35 | 0.01 |
| 2 | Jeger RV, 2018, LANCET, V392, P849, DOI 10.1016/S0140-6736(18)31719-7[2] | 2018 | 29 | 0.02 |
| 3 | Vos NS, 2019, JACC-CARDIOVASC INTE, V12, P1691, DOI 10.1016/j.jcin.2019.04.016[3] | 2019 | 27 | 0.1 |
| 4 | Rissanen TT, 2019, LANCET, V394, P230, DOI 10.1016/S0140-6736(19)31126-2[4] | 2019 | 24 | 0.13 |
| 5 | Jeger RV, 2020, LANCET, V396, P1504, DOI 10.1016/S0140-6736(20)32173-5[5] | 2020 | 21 | 0.01 |
| 6 | Cortese B, 2020, JACC-CARDIOVASC INTE, V13, P2840, DOI 10.1016/j.jcin.2020.08.035[6] | 2020 | 20 | 0.03 |
| 7 | Neumann FJ, 2019, EUR HEART J, V40, P79, DOI 10.1093/eurheartj/ehy855[7] | 2019 | 20 | 0.04 |
| 8 | Ali RM, 2019, JACC-CARDIOVASC INTE, V12, P558, DOI 10.1016/j.jcin.2018.11.040[8] | 2019 | 16 | 0.12 |
| 9 | Tang YD, 2018, JACC-CARDIOVASC INTE, V11, P2381, DOI 10.1016/j.jcin.2018.09.009[9] | 2018 | 16 | 0.03 |
| 10 | Byrne RA, 2013, LANCET, V381, P461, DOI 10.1016/S0140-6736(12)61964-3[10] | 2013 | 14 | 0.05 |

Supplementary Table 7 Top 10 References with the Strongest Citation Bursts.

| References | Year | Strength | Begin | End | 2004 - 2025 |
| --- | --- | --- | --- | --- | --- |
| [Unverdorben M, 2009, CIRCULATION, V119, P2986, DOI 10.1161/CIRCULATIONAHA.108.839282,](http://dx.doi.org/10.1161/CIRCULATIONAHA.108.839282) [11] | 2009 | 5 | 2010 | 2014 | ▂▂▂▂▂▂▃▃▃▃▃▂▂▂▂▂▂▂▂▂▂▂ |
| [Scheller B, 2008, CLIN RES CARDIOL, V97, P773, DOI 10.1007/s00392-008-0682-5,](http://dx.doi.org/10.1007/s00392-008-0682-5) [12] | 2008 | 4.53 | 2011 | 2013 | ▂▂▂▂▂▂▂▃▃▃▂▂▂▂▂▂▂▂▂▂▂▂ |
| [Mathey DG, 2011, EUROINTERVENTION, V7, PK61, DOI 10.4244/EIJV7SKA11,](http://dx.doi.org/10.4244/EIJV7SKA11) [13] | 2011 | 4.26 | 2011 | 2014 | ▂▂▂▂▂▂▂▃▃▃▃▂▂▂▂▂▂▂▂▂▂▂ |
| [Ali RM, 2011, EUROINTERVENTION, V7, PK83, DOI 10.4244/EIJV7SKA15,](http://dx.doi.org/10.4244/EIJV7SKA15) [14] | 2011 | 4.65 | 2012 | 2016 | ▂▂▂▂▂▂▂▂▃▃▃▃▃▂▂▂▂▂▂▂▂▂ |
| [Wöhrle J, 2011, HEART, V97, P1338, DOI 10.1136/hrt.2011.226563,](http://dx.doi.org/10.1136/hrt.2011.226563) [15] | 2011 | 3.58 | 2012 | 2013 | ▂▂▂▂▂▂▂▂▃▃▂▂▂▂▂▂▂▂▂▂▂▂ |
| [Unverdorben M, 2010, CLIN RES CARDIOL, V99, P165, DOI 10.1007/s00392-009-0101-6,](http://dx.doi.org/10.1007/s00392-009-0101-6) [16] | 2010 | 3.58 | 2012 | 2013 | ▂▂▂▂▂▂▂▂▃▃▂▂▂▂▂▂▂▂▂▂▂▂ |
| [Byrne RA, 2013, LANCET, V381, P461, DOI 10.1016/S0140-6736(12)61964-3,](http://dx.doi.org/10.1016/S0140-6736(12)61964-3" \o "http://dx.doi.org/10.1016/S0140-6736(12)61964-3) [10] | 2013 | 6.35 | 2013 | 2018 | ▂▂▂▂▂▂▂▂▂▃▃▃▃▃▃▂▂▂▂▂▂▂ |
| [Latib A, 2012, J AM COLL CARDIOL, V60, P2473, DOI 10.1016/j.jacc.2012.09.020,](http://dx.doi.org/10.1016/j.jacc.2012.09.020) [17] | 2012 | 4.14 | 2013 | 2017 | ▂▂▂▂▂▂▂▂▂▃▃▃▃▃▂▂▂▂▂▂▂▂ |
| [Rittger H, 2012, J AM COLL CARDIOL, V59, P1377, DOI 10.1016/j.jacc.2012.01.015,](http://dx.doi.org/10.1016/j.jacc.2012.01.015) [18] | 2012 | 3.82 | 2013 | 2016 | ▂▂▂▂▂▂▂▂▂▃▃▃▃▂▂▂▂▂▂▂▂▂ |
| [Task Force on Myocardial Revas ...... 2010, EUR HEART J,DOI 10.1093/eurheartj/ehq277,](http://dx.doi.org/10.1093/eurheartj/ehq277)[19] | 2010 | 3.55 | 2013 | 2014 | ▂▂▂▂▂▂▂▂▂▃▃▂▂▂▂▂▂▂▂▂▂▂ |


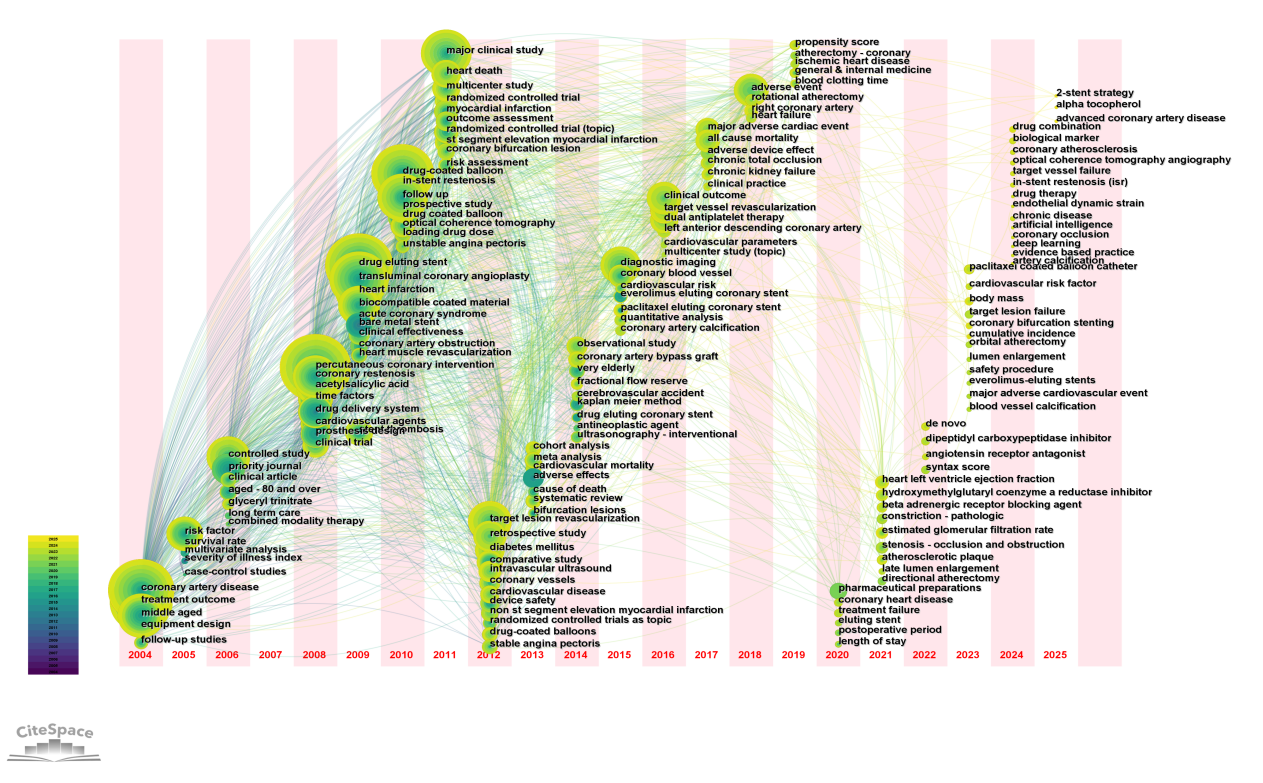


Supplementary Figure 7 Timezone map of keyword evolution in drug-coated balloon DCB research for coronary artery disease.

Supplementary Table 8 Top 20 keywords by frequency of occurrence in DCB research for coronary artery disease.

| Rank | Keyword | Year | Centrality | Count |
| --- | --- | --- | --- | --- |
| 1 | percutaneous coronary intervention | 2008 | 0 | 633 |
| 2 | drug eluting stent | 2009 | 0.02 | 548 |
| 3 | coronary artery disease | 2004 | 0.05 | 536 |
| 4 | treatment outcome | 2008 | 0.69 | 519 |
| 5 | transluminal coronary angioplasty | 2009 | 0.23 | 468 |
| 6 | drug-coated balloon | 2011 | 0 | 445 |
| 7 | in-stent restenosis | 2010 | 0.08 | 380 |
| 8 | major clinical study | 2012 | 0.33 | 344 |
| 9 | follow up | 2011 | 0.02 | 343 |
| 10 | middle aged | 2005 | 0.46 | 325 |
| 11 | heart infarction | 2011 | 0.06 | 307 |
| 12 | coronary restenosis | 2008 | 0.1 | 300 |
| 13 | controlled study | 2009 | 0.13 | 281 |
| 14 | diagnostic imaging | 2016 | 0.02 | 222 |
| 15 | target lesion revascularization | 2016 | 0 | 221 |
| 16 | biocompatible coated material | 2011 | 0.15 | 214 |
| 17 | acetylsalicylic acid | 2008 | 0.4 | 196 |
| 18 | risk factor | 2011 | 0.05 | 193 |
| 19 | prospective study | 2011 | 0.03 | 183 |
| 20 | priority journal | 2010 | 0 | 173 |

Supplementary Table 9 Top 20 Keywords with the Strongest Citation Bursts.

| Keywords | Year | Strength | Begin | End | 2004 - 2025 |
| --- | --- | --- | --- | --- | --- |
| equipment design | 2004 | 14.77 | **2004** | 2013 | ▃▃▃▃▃▃▃▃▃▃▂▂▂▂▂▂▂▂▂▂▂▂ |
| follow-up studies | 2004 | 7.74 | **2004** | 2014 | ▃▃▃▃▃▃▃▃▃▃▃▂▂▂▂▂▂▂▂▂▂▂ |
| prosthesis design | 2008 | 12.42 | **2008** | 2019 | ▂▂▂▂▃▃▃▃▃▃▃▃▃▃▃▃▂▂▂▂▂▂ |
| drug delivery system | 2008 | 11.55 | **2008** | 2017 | ▂▂▂▂▃▃▃▃▃▃▃▃▃▃▂▂▂▂▂▂▂▂ |
| bare metal stent | 2009 | 37.75 | **2009** | 2017 | ▂▂▂▂▂▃▃▃▃▃▃▃▃▃▂▂▂▂▂▂▂▂ |
| drug efficacy | 2009 | 6.96 | **2009** | 2013 | ▂▂▂▂▂▃▃▃▃▃▂▂▂▂▂▂▂▂▂▂▂▂ |
| drug coated balloon | 2010 | 29.56 | **2010** | 2017 | ▂▂▂▂▂▂▃▃▃▃▃▃▃▃▂▂▂▂▂▂▂▂ |
| coronary stent | 2011 | 9.74 | **2011** | 2014 | ▂▂▂▂▂▂▂▃▃▃▃▂▂▂▂▂▂▂▂▂▂▂ |
| drug delivery systems | 2011 | 7.37 | **2011** | 2014 | ▂▂▂▂▂▂▂▃▃▃▃▂▂▂▂▂▂▂▂▂▂▂ |
| priority journal | 2009 | 21.8 | **2012** | 2020 | ▂▂▂▂▂▂▂▂▃▃▃▃▃▃▃▃▃▂▂▂▂▂ |
| heart disease | 2012 | 11.45 | **2012** | 2016 | ▂▂▂▂▂▂▂▂▃▃▃▃▃▂▂▂▂▂▂▂▂▂ |
| coronary artery recanalization | 2012 | 11.37 | **2012** | 2016 | ▂▂▂▂▂▂▂▂▃▃▃▃▃▂▂▂▂▂▂▂▂▂ |
| kaplan-meier estimate | 2011 | 8.31 | **2013** | 2017 | ▂▂▂▂▂▂▂▂▂▃▃▃▃▃▂▂▂▂▂▂▂▂ |
| adverse effects | 2013 | 22.67 | **2014** | 2017 | ▂▂▂▂▂▂▂▂▂▂▃▃▃▃▂▂▂▂▂▂▂▂ |
| percutaneous transluminal angioplasty balloon | 2014 | 8.3 | **2014** | 2017 | ▂▂▂▂▂▂▂▂▂▂▃▃▃▃▂▂▂▂▂▂▂▂ |
| very elderly | 2014 | 7.56 | **2014** | 2019 | ▂▂▂▂▂▂▂▂▂▂▃▃▃▃▃▃▂▂▂▂▂▂ |
| ptca catheter | 2014 | 7.38 | **2014** | 2017 | ▂▂▂▂▂▂▂▂▂▂▃▃▃▃▂▂▂▂▂▂▂▂ |
| everolimus eluting coronary stent | 2015 | 12.11 | **2015** | 2017 | ▂▂▂▂▂▂▂▂▂▂▂▃▃▃▂▂▂▂▂▂▂▂ |
| adverse event | 2018 | 19.67 | **2019** | 2022 | ▂▂▂▂▂▂▂▂▂▂▂▂▂▂▂▃▃▃▃▂▂▂ |
| pharmaceutical preparations | 2020 | 16.41 | **2020** | 2021 | ▂▂▂▂▂▂▂▂▂▂▂▂▂▂▂▂▃▃▂▂▂▂ |

1. Jeger RV, Eccleshall S, Wan Ahmad WA, Ge J, Poerner TC, Shin ES, Alfonso F, Latib A, Ong PJ, Rissanen TT *et al*: **Drug-Coated Balloons for Coronary Artery Disease: Third Report of the International DCB Consensus Group**. *JACC Cardiovasc Interv* 2020, **13**(12):1391-1402.

2. Jeger RV, Farah A, Ohlow MA, Mangner N, Mobius-Winkler S, Leibundgut G, Weilenmann D, Wohrle J, Richter S, Schreiber M *et al*: **Drug-coated balloons for small coronary artery disease (BASKET-SMALL 2): an open-label randomised non-inferiority trial**. *Lancet* 2018, **392**(10150):849-856.

3. Vos NS, Fagel ND, Amoroso G, Herrman JR, Patterson MS, Piers LH, van der Schaaf RJ, Slagboom T, Vink MA: **Paclitaxel-Coated Balloon Angioplasty Versus Drug-Eluting Stent in Acute Myocardial Infarction: The REVELATION Randomized Trial**. *JACC Cardiovasc Interv* 2019, **12**(17):1691-1699.

4. Rissanen TT, Uskela S, Eranen J, Mantyla P, Olli A, Romppanen H, Siljander A, Pietila M, Minkkinen MJ, Tervo J *et al*: **Drug-coated balloon for treatment of de-novo coronary artery lesions in patients with high bleeding risk (DEBUT): a single-blind, randomised, non-inferiority trial**. *Lancet* 2019, **394**(10194):230-239.

5. Jeger RV, Farah A, Ohlow MA, Mangner N, Mobius-Winkler S, Weilenmann D, Wohrle J, Stachel G, Markovic S, Leibundgut G *et al*: **Long-term efficacy and safety of drug-coated balloons versus drug-eluting stents for small coronary artery disease (BASKET-SMALL 2): 3-year follow-up of a randomised, non-inferiority trial**. *Lancet* 2020, **396**(10261):1504-1510.

6. Cortese B, Di Palma G, Guimaraes MG, Piraino D, Orrego PS, Buccheri D, Rivero F, Perotto A, Zambelli G, Alfonso F: **Drug-Coated Balloon Versus Drug-Eluting Stent for Small Coronary Vessel Disease: PICCOLETO II Randomized Clinical Trial**. *JACC Cardiovasc Interv* 2020, **13**(24):2840-2849.

7. Neumann FJ, Sousa-Uva M: **'Ten commandments' for the 2018 ESC/EACTS Guidelines on Myocardial Revascularization**. *Eur Heart J* 2019, **40**(2):79-80.

8. Ali RM, Abdul Kader M, Wan Ahmad WA, Ong TK, Liew HB, Omar AF, Mahmood Zuhdi AS, Nuruddin AA, Schnorr B, Scheller B: **Treatment of Coronary Drug-Eluting Stent Restenosis by a Sirolimus- or Paclitaxel-Coated Balloon**. *JACC Cardiovasc Interv* 2019, **12**(6):558-566.

9. Tang Y, Qiao S, Su X, Chen Y, Jin Z, Chen H, Xu B, Kong X, Pang W, Liu Y *et al*: **Drug-Coated Balloon Versus Drug-Eluting Stent for Small-Vessel Disease: The RESTORE SVD China Randomized Trial**. *JACC Cardiovasc Interv* 2018, **11**(23):2381-2392.

10. Byrne RA, Neumann FJ, Mehilli J, Pinieck S, Wolff B, Tiroch K, Schulz S, Fusaro M, Ott I, Ibrahim T *et al*: **Paclitaxel-eluting balloons, paclitaxel-eluting stents, and balloon angioplasty in patients with restenosis after implantation of a drug-eluting stent (ISAR-DESIRE 3): a randomised, open-label trial**. *Lancet* 2013, **381**(9865):461-467.

11. Unverdorben M, Vallbracht C, Cremers B, Heuer H, Hengstenberg C, Maikowski C, Werner GS, Antoni D, Kleber FX, Bocksch W *et al*: **Paclitaxel-coated balloon catheter versus paclitaxel-coated stent for the treatment of coronary in-stent restenosis**. *Circulation* 2009, **119**(23):2986-2994.

12. Scheller B, Hehrlein C, Bocksch W, Rutsch W, Haghi D, Dietz U, Böhm M, Speck U: **Two year follow-up after treatment of coronary in-stent restenosis with a paclitaxel-coated balloon catheter**. *Clin Res Cardiol* 2008, **97**(10):773-781.

13. Mathey DG, Wendig I, Boxberger M, Bonaventura K, Kleber FX: **Treatment of bifurcation lesions with a drug-eluting balloon: the PEPCAD V (Paclitaxel Eluting PTCA Balloon in Coronary Artery Disease) trial**. *EuroIntervention* 2011, **7 Suppl K**:K61-65.

14. Ali RM, Degenhardt R, Zambahari R, Tresukosol D, Ahmad WA, Kamar H, Kui-Hian S, Ong TK, bin Ismail O, bin Elis S *et al*: **Paclitaxel-eluting balloon angioplasty and cobalt-chromium stents versus conventional angioplasty and paclitaxel-eluting stents in the treatment of native coronary artery stenoses in patients with diabetes mellitus**. *EuroIntervention* 2011, **7 Suppl K**:K83-92.

15. Wohrle J, Birkemeyer R, Markovic S, Nguyen TV, Sinha A, Miljak T, Spiess J, Rottbauer W, Rittger H: **Prospective randomised trial evaluating a paclitaxel-coated balloon in patients treated with endothelial progenitor cell capturing stents for de novo coronary artery disease**. *Heart* 2011, **97**(16):1338-1342.

16. Unverdorben M, Kleber FX, Heuer H, Figulla HR, Vallbracht C, Leschke M, Cremers B, Hardt S, Buerke M, Ackermann H *et al*: **Treatment of small coronary arteries with a paclitaxel-coated balloon catheter**. *Clin Res Cardiol* 2010, **99**(3):165-174.

17. Latib A, Colombo A, Castriota F, Micari A, Cremonesi A, De Felice F, Marchese A, Tespili M, Presbitero P, Sgueglia GA *et al*: **A randomized multicenter study comparing a paclitaxel drug-eluting balloon with a paclitaxel-eluting stent in small coronary vessels: the BELLO (Balloon Elution and Late Loss Optimization) study**. *J Am Coll Cardiol* 2012, **60**(24):2473-2480.

18. Rittger H, Brachmann J, Sinha AM, Waliszewski M, Ohlow M, Brugger A, Thiele H, Birkemeyer R, Kurowski V, Breithardt OA *et al*: **A randomized, multicenter, single-blinded trial comparing paclitaxel-coated balloon angioplasty with plain balloon angioplasty in drug-eluting stent restenosis: the PEPCAD-DES study**. *J Am Coll Cardiol* 2012, **59**(15):1377-1382.

19. Task Force on Myocardial Revascularization of the European Society of C, the European Association for Cardio-Thoracic S, European Association for Percutaneous Cardiovascular I, Wijns W, Kolh P, Danchin N, Di Mario C, Falk V, Folliguet T, Garg S *et al*: **Guidelines on myocardial revascularization**. *Eur Heart J* 2010, **31**(20):2501-2555.
